# Global patterns of parasite sharing among freshwater turtles

**DOI:** 10.1007/s00436-026-08702-5

**Published:** 2026-06-02

**Authors:** Maria Silva, João Rato, Pedro Brandão, Rita Rocha, Pedro Anastácio, Filipe Banha

**Affiliations:** 1 MARE – Marine and Environmental Sciences Centre/ARNET - Aquatic Research Network, Universidade de Évora, Évora, Portugal; 2 IIFA - Instituto de Investigação e Formação Avançada, Universidade de Évora, Palácio do Vimioso, Largo Marquês de Marialva, Apartado 94, Évora, 7002-554 Portugal; 3 BIOPOLIS Program in Genomics, Biodiversity and Land Planning, CIBIO- InBIO, University of Porto, Vairão Campus, Rua Padre Armando Quintas, Vairão, 4485-661 Portugal; 4 Veterinary Hospital, Universidade de Évora, Mitra Campus, Apartado 94, Évora, 7002-554 Portugal; 5 Department of Landscape, Environment, and Planning, Universidade de Évora, Rua Romão Ramalho 59, Évora, 7000-671 Portugal

**Keywords:** Terrapins, Parasite, Invasive species, Host–parasite, One health, Conservation

## Abstract

Freshwater turtles represent a highly diverse and ecologically important group, yet they are among the most threatened vertebrates worldwide due to habitat loss, overexploitation, and the introduction of non-native species. In this study, we conducted a systematic literature review, following PRISMA guidelines, to assess parasite sharing among native and non-native freshwater turtles globally, compiling data from 294 publications spanning 1939 to 2025. Our analyses, including multiple correspondence analysis (MCA) and clustering analysis, revealed that parasite communities are strongly structured by host phylogeny, biogeography, and the presence of non-native species. In North America, Asia, and South America, both native and introduced turtles share extensive parasite networks, whereas Oceania and Africa show mostly endemic, intra-native associations. Non-native species such as *Trachemys scripta* may act as epidemiological bridges in Europe, facilitating parasite spillover to native hosts. These findings underscore the influence of evolutionary history, species introductions, and geographic isolation on host–parasite dynamics and highlight the need for targeted monitoring and management strategies to mitigate ecological and health risks.

**Supplementary Information:**

The online version contains supplementary material available at 10.1007/s00436-026-08702-5.

## Introduction

Turtles are an ancient, widely distributed, and easily recognizable group, playing significant roles across various cultures and possessing high medicinal and economic value on a global scale (Bárcenas-García et al. 2022). Freshwater turtles represent a substantial portion of chelonian diversity, comprising approximately 81% of the more than 360 described species (Uetz et al. 2025). Although restricted to roughly 1% of the Earth’s surface area, freshwater systems support communities of these animals with broad geographic distribution, being absent only from the Antarctic continent (Bour 2008). Freshwater turtles are extensively exploited as a food resource, with their meat and eggs being consumed in many regions (Stanford et al. 2020). Additionally, their fat, viscera, and shells are used for various purposes, including traditional medicine (Pezzuti et al. 2010; Bárcenas-García et al. 2022). Finally, turtles are one of the most important groups in the international pet trade (Dudgeon 2019; Sigouin et al. 2017). Despite their importance to human societies (Stanford et al. 2020) and the equilibrium of aquatic ecosystems (Moll and Moll 2004; Lovich et al. 2018), according to the IUCN Red List, freshwater turtles already represent one of the most threatened group of vertebrates (Rhodin et al. 2018; Stanford et al. 2020; Tickner et al. 2020). Even within protected areas, these animals remain vulnerable to anthropogenic impacts (Howell et al. 2019; Norris et al. 2019).

The introduction of species outside their native ranges is currently regarded as one of the leading threats to biodiversity, particularly on islands (Bellard et al. 2022). In this context, non-native species can negatively affect native species through predation, hybridization, pathogen transmission, or competition for resources (Polo-Cavia et al. 2009). For instance, the red-eared slider *Trachemys scripta* (Thunberg, 1792) is one of the most recognized examples of a non-native species, known to compete for essential resources with native species (Pleguezuelos 2002; Cadi and Joly 2004). Beyond direct competition, additional concerns have been raised regarding the role of *T. scripta* as a vector of parasites. Documented cases include host-switching of endoparasitic flatworms, as well as infections by co-introduced helminths, with serious implications for the health of native populations (Demkowska-Kutrzepa et al. 2018; Rato et al. 2024). Parasite diversity in freshwater turtles varies considerably across different families and species, being influenced by factors such as geographic distribution, invasive status, and life history traits (Rato et al. 2024). The family Emydidae has been extensively studied, with a substantial number of publications focusing on *T. scripta* and *Emys orbicularis* (Linnaeus, 1758), revealing a wide array of pathogenic agents (Rato et al. 2024).

The present study aims to identify parasite sharing among freshwater turtle species. Specifically, we seek to detect which parasites are potentially shared between native and non-native species, and their geographic and phylogenetic patterns. While host–parasite associations in freshwater turtles have been documented, relatively few studies have explored these interactions using a comparative framework across species and regions. By applying multivariate exploratory analyses, namely multiple correspondence analysis (MCA), we are able to evaluate patterns of parasite sharing in a systematic and integrative way that has rarely been done in previous research. Such information is essential for the development and refinement of health assessment protocols, epidemiological monitoring programs, conservation initiatives, and one health strategies. Additionally, this study aims to identify knowledge gaps concerning both host species and under-studied geographic regions and parasitic groups.

## Methodology

For our literature review, we followed the approach and dataset previously published in Rato et al. (2024), updating it with articles published during 2024 and adhering to PRISMA guidelines (Veroniki et al. 2025). For this updated search, we retrieved publications from Google Scholar, Web of Science, and Scopus using a series of keyword combinations (Supplementary Materials 1). We excluded articles that were not published in peer-reviewed journals, were not written in English, did not include freshwater turtle hosts, or did not specify at least the host genus. We also excluded studies in which diseases were not caused by parasites or where the parasite has not been identified; or where the parasite could not be taxonomically identified to at least the genus level. Additionally, we excluded studies that lacked information on geographic origin, making it impossible to determine whether hosts were native or non-native. Countries that overlap with any part of a species’ native range were considered native-range countries. For each study, we extracted information on the host (species and native/non-native status), the parasite (genus and, when possible, species), and the continent where the parasite was reported. We included studies conducted both in the wild and in captivity, since captive animals may pose a health risk to humans and can potentially escape or be released, creating risks for local wildlife.

Next, we conducted a multiple correspondence analysis (MCA) (Abdi and Valentin 2007), based on parasite associations to evaluate which freshwater turtle (FT) populations, both native and non-native, shared the greatest number of parasites. To identify patterns associated with different endoparasite groups (1 –Trematoda, 2 - Monogenea and Cestoda [Platyhelminthes]; 3 – Protozoa and Chromista; 4 - Nematoda and Acanthocephala) and ectoparasites (5) we performed separate MCA analyses for each group. The data used for these analyses were categorized by continent (Europe, North America, South America, Asia, Oceania, and Africa), host species (freshwater turtle species), and nativeness (native or non-native). We used Jaccard’s distance because many studies report only the presence of a parasite genus without quantifying infection levels, and this metric is well-suited for our binary type data (Choi et al., 2010).

A circular dendrogram was then plotted to visualize the hierarchical clustering of parasites shared among hosts by continent. This visualization provided an intuitive representation of the relationships among host populations and their shared parasites, highlighting similarities and differences across continents and species.

All statistical analyses were performed using the MASS package (Venables and Ripley, 2002) in RStudio (Version 1.1.463;R Core Team, 2022), with a significance threshold of *p* < 0.05.

## Results

### Temporal and spatial trends of parasite records

**Fig. 1:**
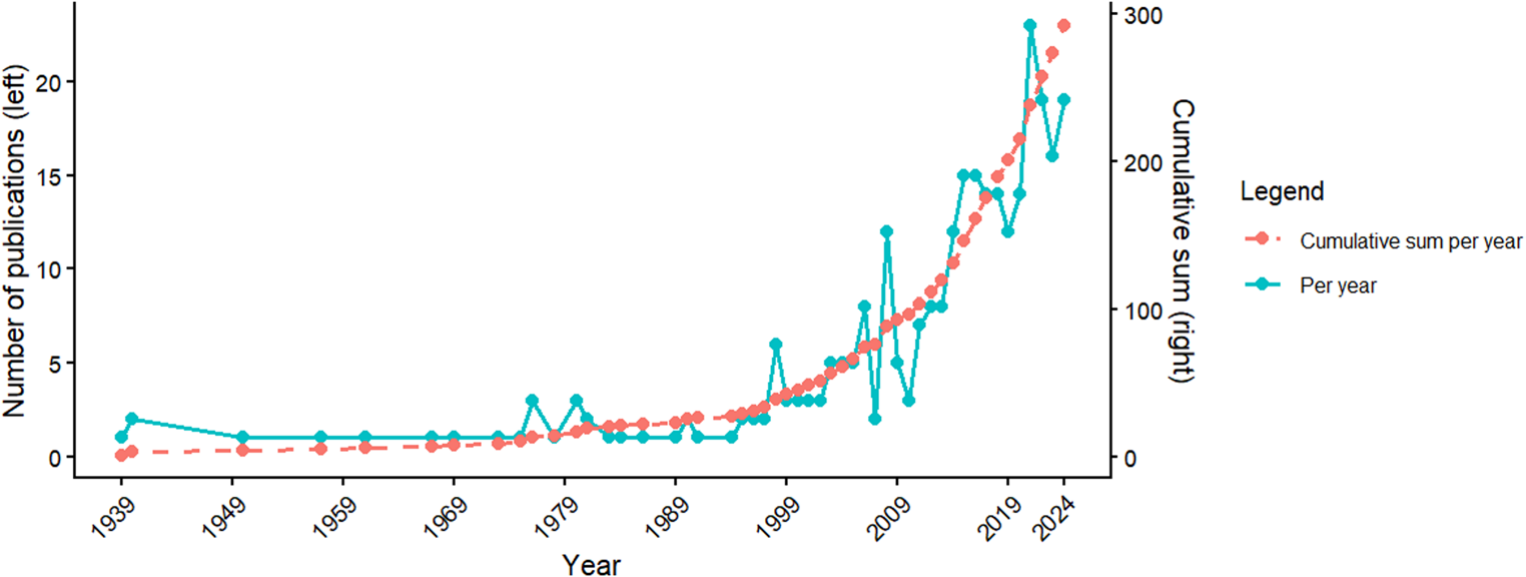
Annual and cumulative distribution of publications on parasites in freshwater turtles between 1939 and 2024

A total of 294 scientific articles were analyzed in the present review, all of which reported infections in freshwater chelonians caused by parasites. In these studies, the parasites were identified for each host species (turtles), allowing for a detailed characterization of host–parasite relationships. The compiled information covers the period between 1939 and June 2025 (which is not included in the graph, excluding the 2025 data as it only represents six months rather than the full year and is therefore not sufficiently representative) (Fig. 1), with the most recent study to date being that of Gurbanova (2025).

The temporal analysis reveals a marked increase in the number of publications over the decades. As illustrated in Fig. 1, the number of articles published per year remained low and relatively stable until the late 1970 s, with annual values rarely exceeding five publications. From the 1980 s onwards, a gradual increase in publication frequency was observed, followed by a pronounced acceleration after the 2000s.

**Fig. 2:**
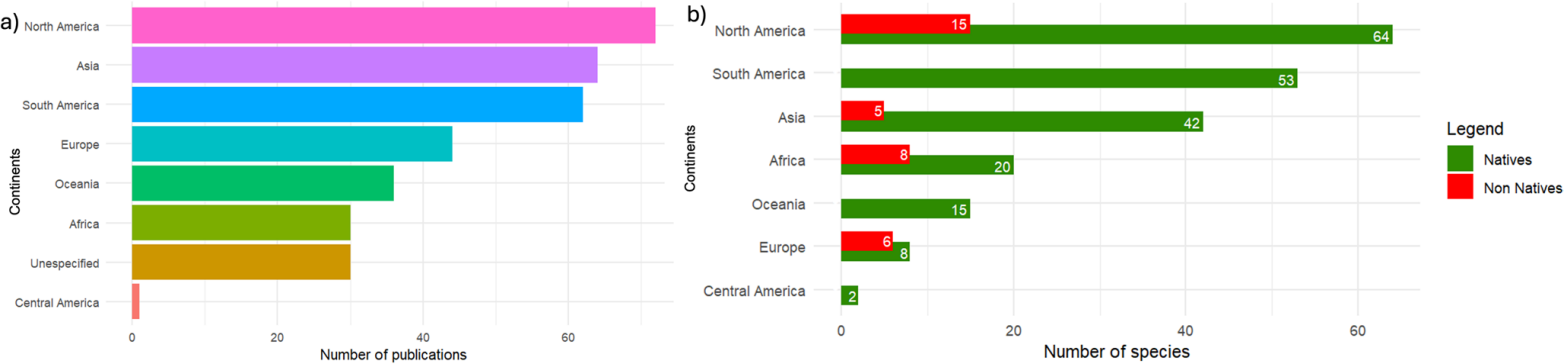
(**a**) Geographical distribution of publications on parasites in freshwater turtles by continent, including those without specified origin (classified as unspecified). (**b**) Geographical distribution of freshwater turtle species described with parasites by continent. The Americas are presented separately as North America, Central America, and South America

The analysis of the geographical distribution of publications reporting parasite species in FT (Fig. 2a) reveals a clear predominance of North America, particularly the United States (*n* = 62), which overwhelmingly leads scientific production in this field. Asia and South America also show strong representation, with Malaysia (*n* = 12), Japan (*n* = 10), and India (*n* = 9) contributing substantially, while Brazil (*n* = 38) and Argentina (*n* = 7) stand out within the South American context.

Europe likewise exhibits a noteworthy level of research activity, mainly driven by Spain (*n* = 17), France (*n* = 7), and Italy (*n* = 5). Smaller contributions originate from Oceania, primarily Australia (*n* = 31), and from Africa, represented by Algeria (*n* = 6), Morocco (*n* = 6), Tunisia (*n* = 4), and Nigeria (*n* = 4).

The distribution of native and non-native turtle species with parasite records across continents (Fig. 2b) reveals regional differences. Across all continents, most of the turtle diversity associated with parasites is composed of native species. Non-native species occur in lower numbers and are mainly concentrated in North America, Europe, and Africa.

North America exhibited the highest richness of native turtle species with parasite records (*n* = 64), in addition to a substantial number of non-native species (*n* = 15). South America showed exclusively native species (*n* = 53), with no records of non-native species associated with parasites. A similar pattern was observed in Oceania, where all 15 recorded species are native. In Asia, 42 native species with parasites were recorded, along with only 5 non-native species. Africa exhibited 20 native and 8 non-native species, indicating a relatively higher proportion of introduced species compared to most other continents. Europe showed low overall richness, with 8 native and 6 non-native species, reflecting a more balanced proportion between native and introduced taxa. Finally, Central America presented the lowest richness, with only 2 native species and no records of non-native species. *Trachemys scripta* was the most frequently reported species with parasite records (*n* = 136), followed by *E. orbicularis* (*n* = 104).

### Host analysis

**Fig. 3:**
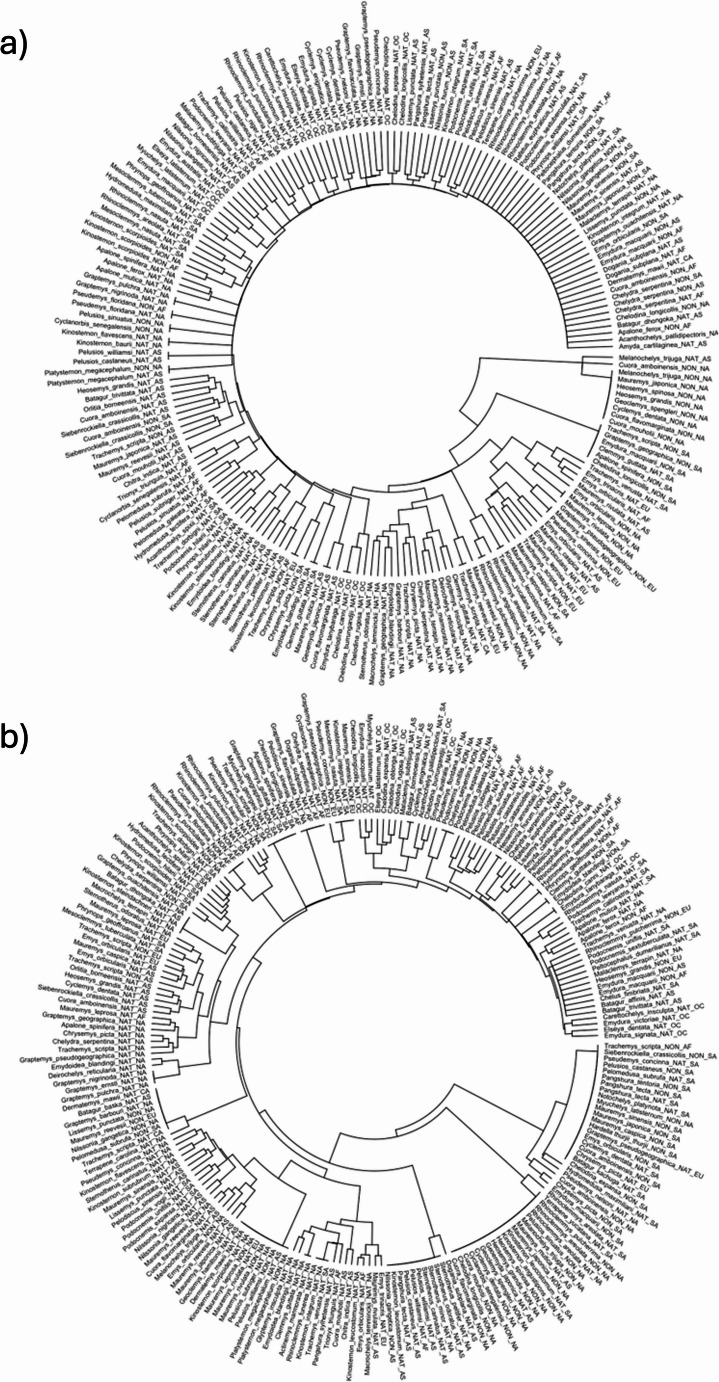
Parasite clustering among freshwater turtle species at the global level based on similarity of parasites: (**a**) based on the presence of shared parasite species and (**b**) based on the presence of shared genera. Population coding follows the format species_NAT/NON_continent, where “species” is the host species, “NAT” denotes native species and “NON” denotes non-native species, followed by the abbreviation of the continent of occurrence

In our dataset, the most predominant host genera were *Trachemys* and *Mauremys*, while *Cycloderma*, *Geoemyda*, *Hardella*, and *Rafetus* were among the least represented. *T. scripta* was associated with multiple regions and was the most frequently reported species, followed by *E. orbicularis*. Overall, native species were more commonly described (Fig. 3).

Closely related genera such as *Trachemys*, *Graptemys*, and *Pseudemys* exhibited a high degree of parasite overlap. This pattern was also observed in non-native regions, where these genera continued to share many of the same parasite taxa. In contrast, genera that are phylogenetically more distant, such as *Pelomedusa* and *Pelusios*, showed consistently lower parasite overlap with the North American turtles from the Emydidae family, both within their native ranges and when introduced elsewhere. North American species, including *Chelydra serpentina* (Linnaeus, 1758) and *Graptemys pseudogeographica* (Gray, 1831), displayed greater intra-continental parasite sharing, particularly with other North American genera such as *Trachemys*, *Graptemys*, and *Pseudemys*, whereas species from Africa and Asia exhibited more distinct and region-specific patterns. In contrast, FT species from Oceania showed a clear separation in parasite composition compared to genera and species from other continents. Genera such as *Emydura* and *Elseya*, which occur exclusively in Oceania, did not share parasites with other turtle groups.

Native species from the same region tend to host similar parasites. Conversely, non-native species often harbour parasites both from their native ranges and from the areas into which they have been introduced. However, it is also necessary to consider that some of these parasites may not be intrinsically associated with the invasive species, but may instead result from accidental introduction via carrier hosts or through contact with novel biological communities. The cluster analysis reveals a strong association between parasite sharing of freshwater turtle genera, with distinct patterns observed between native (NAT) and non-native (NON) species (Fig. 3).

## Geographic patterns/analysis

### Europe

**Fig. 4:**
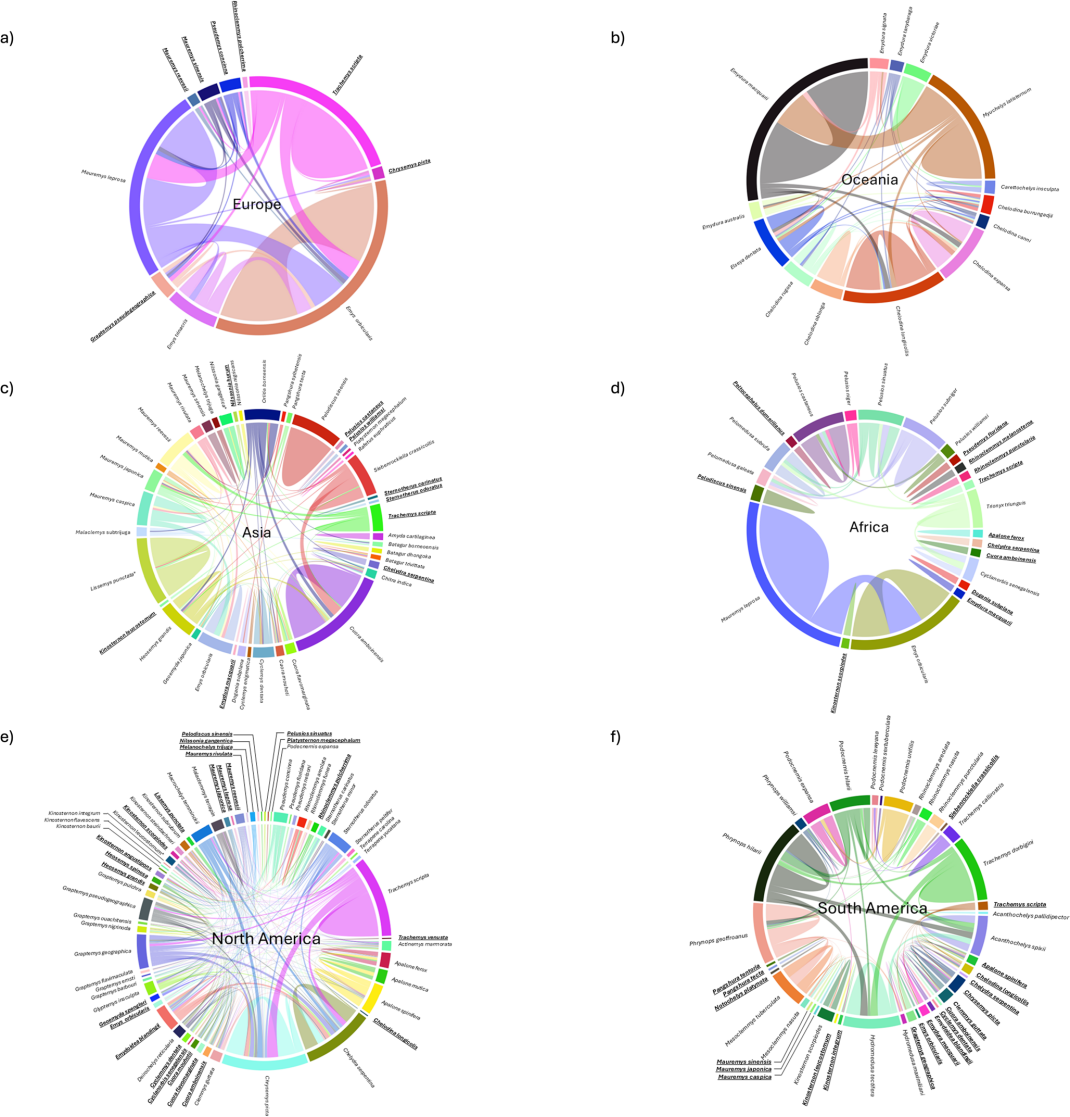
(**a**) Graphical representation of the sharing of parasites among the freshwater turtle species documented in the literature for Europe. Non-native species are indicated in bold and underlined. (**b**) Graphical representation of the sharing of parasites among the freshwater turtle species documented in the literature for Oceania. (**c**) Graphical representation of the sharing of parasites among the freshwater turtle species documented in the literature for Asia. Non-native species are indicated in bold and underlined. * had places where they are native and non-native. (**d**) Graphical representation of the description and sharing of parasites among the freshwater turtle species documented in the literature for Africa. Non-native species are indicated in bold and underlined. (**e**) Graphical representation of the sharing of parasites among the freshwater turtle species documented in the literature for North America. Non-native species are indicated in bold and underlined. * had places where they are native and non-native. (**f**) Graphical representation of the sharing of parasites among the freshwater turtle species documented in the literature for South America. Non-native species are indicated in bold and underlined

On the European continent, the three native species *M. leprosa*, *E. orbicularis* and *Emys trinacris* (Fritz et al., 2005) share parasites between them as well as with the non-native species *T. scripta* (Fig. 4a)).

Among the non-native species, *G. pseudogeographica* shares parasites with two native species, *E. trinacris* and *M. leprosa*. *Chrysemys picta* (Schneider, 1783) also displays parasitic associations with the non-native *T. scripta*, but additionally with the native *M. leprosa* and *E. orbicularis*. Notably, *T. scripta*, one of the most widely distributed non-native turtles in Europe, shares parasites with all represented species, both native and non-native, except *E. trinacris*. Overall, Europe exhibits substantial parasite sharing between native and non-native FT, in particular, *G. pseudogeographica* establishes parasitic associations with the native *M. leprosa* and *E. trinacris*. Conversely, the opposite pattern is also observed, as non-native species such as *C. picta* and *T. scripta* share parasites both with each other and with several native species.

### Oceania

In Oceania, only native species with parasites are reported (Fig. 4b)), where *Emydura macquarii* (Gray, 1830) is observed to share parasites with *Emydura australis* (Gray, 1841) and *Elseya dentata* (Gray, 1863). *E. australis* also shares parasites with *E. dentata*.

*E. dentata*, in turn, maintains parasite sharing with *Chelodina expansa* (Gray, 1857). *C. expansa* shares parasites with both *Chelodina canni* (McCord and Thomson, 2002) and *Chelodina burrungandjii* (Thomson, Kennett, and Georges, 2000). *Myuchelys latisternum* (Gray, 1867) also exhibits parasitic interactions with other species, showing the strongest correspondence with *E. macquarii*.

### Asia

Analysis of parasite sharing in Asia revealed a consistent pattern among native turtle species (Fig. 4c)). *Mauremys caspica* (Gmelin, 1774) shares parasites with *M. japonica*, *M. mutica*, and *M. reevesii*. Additionally, species belonging to different families, such as *Malayemys subtrijuga* (Schlegel & Müller, 1845) and *Lissemys punctata* (Bonnaterre, 1789), also demonstrate parasite sharing. Large-bodied riverine species, including *Amyda cartilaginea* (Boddaert, 1770) and several species of the genus *Batagur* (*B. baska*, *B. dhongoka*, and *B. trivittata*), likewise exhibit shared parasitic interactions. Among softshell turtles, *Chitra indica* (Gray, 1831) and *Pelochelys cantorii* (Gray, 1864) also share parasites.

Parasite sharing between non-native and native species is also evident. The non-native *T. scripta* shares parasites with several native Asian species, including *Mauremys reevesii* (Gray, 1831), *M. subtrijuga*, *Sternotherus odoratus* (Latreille in Sonnini & Latreille, 1801), and *Pelodiscus sinensis* (Wiegmann, 1835). *P. sinensis*, although native to part of its range, also exhibits parasitic interactions with introduced species such as *T. scripta* and *Sternotherus carinatus* (Gray, 1856). Finally, *S. carinatus* shares parasites with *S. odoratus*, both non-native species.

### Africa

In Africa, a predominantly intra-native pattern of parasite sharing is observed (Fig. 4 d)). Native species show parasite sharing exclusively among themselves, while non-native species present on the continent exhibit no evidence of parasite sharing, either with native species or among other non-native. Within this framework, the native *M. leprosa* shares parasites only with another native species, *Emys orbicularis*, and the latter shows no parasitic associations with any additional species included in the dataset.

Within the genus *Pelusios*, there is clear overlap of parasites among species, with *Pelusios subniger* (Bonnaterre, 1789) and *Pelusios sinuatus* (Smith, 1838) *also* sharing parasites with both species of *Pelomedusa*. Additionally, a parasitic association is recorded between *Trionyx triunguis* (Forskål, 1775) and *Cyclanorbis senegalensis* (Duméril & Bibron, 1835), both native species.

### North America

The analysis of parasite sharing among North American freshwater turtles (Fig. 4e)) reveals a high level of interconnectivity among the genera *Trachemys*, *Graptemys*, and *Pseudemys*, which share a substantial number of parasite species. *T. scripta* stands out as the species with the highest number of connections and is also the most frequently reported in the literature. *Trachemys venusta* (Gray, 1856) and *Actinemys marmorata* (Baird & Girard, 1852) also show associations with multiple parasite species, indicating a broad parasitic diversity within these taxa.

Species such as *C. serpentina*, *G. pseudogeographica*, and *Apalone mutica* (Lesueur, 1827) likewise exhibit considerable parasite sharing. In contrast, *Emydoidea blandingii* (Holbrook 1838), *Kinosternon flavescens* (Agassiz, 1857), and *S. odoratus* show a more restricted number of parasitic associations. Overall, non-native species (*T. scripta*, *T. venusta*) maintain connections with several parasite genera, sharing many of them with native North American species.

### South America

In South America, the native species *Phrynops hilarii *(Duméril & Bibron, 1835), *Phrynops geoffroanus* (Schweigger, 1812), and *Acanthochelys spixii* (Duméril and Bibron 1835) show the highest number of recorded parasitic associations, sharing several parasite genera among themselves and with other species belonging to the same group. Connections are also observed between *Podocnemis expansa* (Schweigger, 1812) and other South American turtles, although with a more limited range of shared parasites (Fig. 4f)).

Among the non-native species, *T. scripta*, *Mauremys sinensis* (Gray, 1834), *Mauremys japonica* (Temminck and Schlegel 1835), and *M. caspica* are represented, displaying parasitic sharing with several local species. *Hydromedusa tectifera* (Cope, 1870), *Chelus fimbriata* (Schneider, 1783), and *Acanthochelys pallidipectoris* (Freiberg, 1945) also exhibit associations with different parasite groups, although less frequently. Overall, the diagram shows that most species share at least one parasite genus with other turtles present on the continent, reflecting substantial host overlap within the South American freshwater turtle community.

### Global pattern

The analysis of publications spanning from 1939 to 2025 revealed not only a marked temporal increase in research on freshwater turtle parasites, particularly from North America, Europe, and Asia, but also clear geographic and phylogenetic patterns in parasite sharing. Native species tend to harbour region-specific parasites, whereas non-native turtles often carry both native and introduced parasite taxa, facilitating novel host–parasite interactions. Distinct continental patterns emerge: Europe exhibits extensive parasite sharing between native and non-native species, Oceania shows largely endemic associations among native turtles, and Asia, Africa, and the Americas display varied overlaps according to host phylogeny and local species composition. These patterns set the stage for detailed analyses of specific parasite groups, as revealed by Multiple Correspondence Analysis (MCA), highlighting differential associations among Trematoda, Monogenea, Cestoda, Protozoa, Chromista, Nematoda, Acanthocephala, and Ectoparasites, which reflect both evolutionary history and biogeographic constraints of their turtle hosts.

## Parasitic group analysis

### Platyhelminthes: Trematoda

**Fig. 5:**
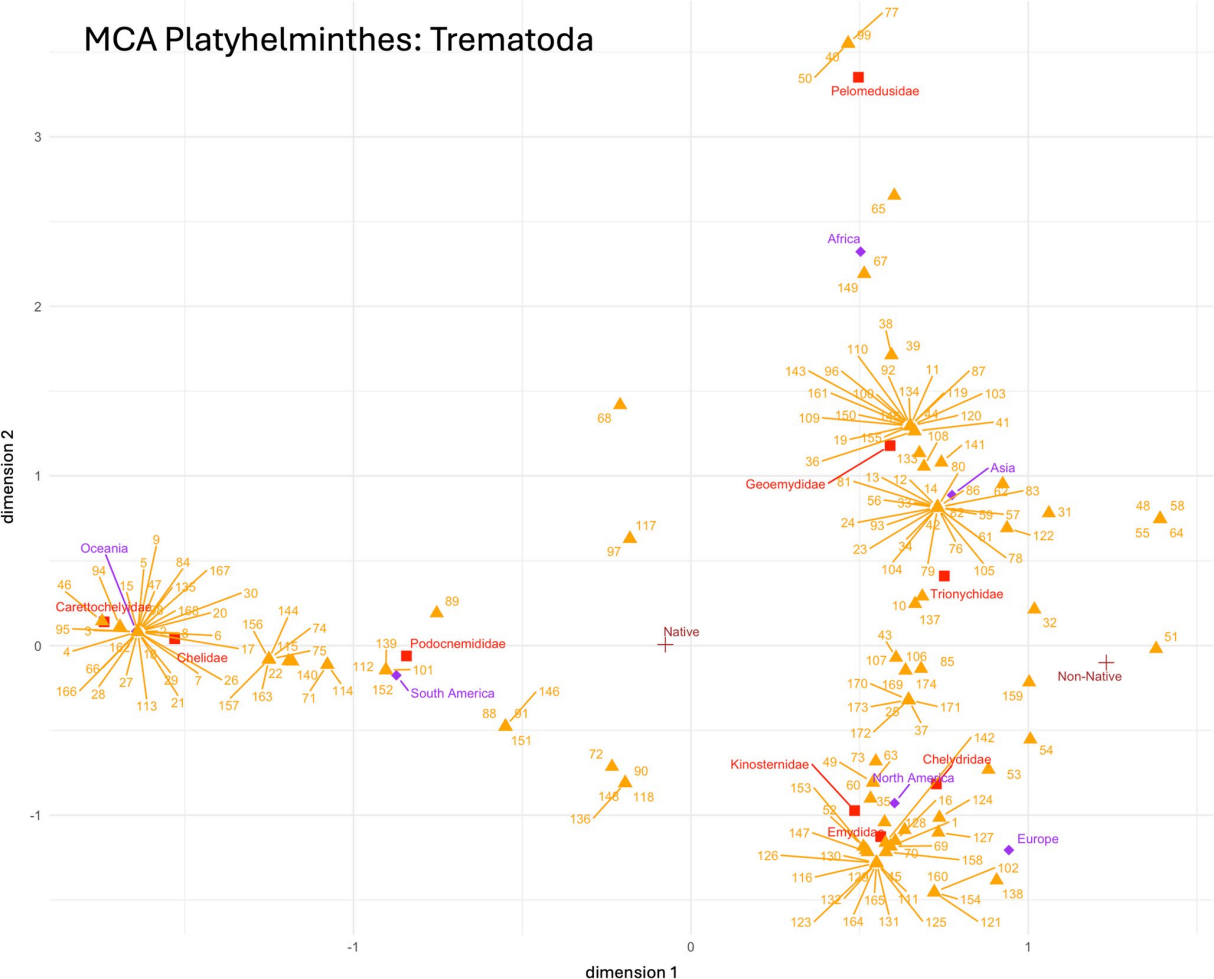
Two-dimensional representation of the results of the Multiple Correspondence Analysis (MCA) applied to the occurrence data of Platyhelminthes, specifically trematodes (numbered variables), in freshwater turtle hosts. Numbers are listed in Supplementary Materials 3. Host species are categorised according to taxonomic family (red), continent of origin (purple), and occurrence status (pink: Native or Non-Native). Legend: Orange numbers correspond to the different trematode morphotypes identified in the analysed turtles; red squares represent the taxonomic families of the turtle hosts; purple diamonds indicate the continents of origin of the host species; pink crosses refer to the occurrence status of the host species within the study area (Native or Non-Native).

The MCA reveals distinct groupings of trematodes according to the phylogeny and biogeographic origin of the turtle hosts (Fig. 5). On the right side of the plot, a strong association is observed between the trematodes *Multicotyle purvisi* (87), *Neopronocephalus orientalis* (92), *Ocadiatrema taiwanense* (96), *Spirhapalum elongatum* (120), and *Stunkardia dilymphosa* (133) and the families Trionychidae and Geoemydidae. These families correspond predominantly to Asian species.

In South America, turtles of the family Podocnemididae are associated with the trematodes *Pitiutrema revelae* (101), *Telorchis bifurcus* (139), and *Telorchis konoi* (152). In North America, shared trematode associations with Europe are observed, involving the families Kinosternidae, Chelydridae, and Emydidae. Within Emydidae, several species-specific trematodes are highlighted, including *Heronimus chelydrae* (69), *Heronimus mollis* (70), *Spirorchis haematobius* (128), and *Telorchis singularis* (158).

Still on the right side, an association with non-native species is observed, including mainly turtle families from Europe and North America, such as Chelydridae, Kinosternidae, and Emydidae, as well as Asian families, namely Geoemydidae and Trionychidae.

In contrast, on the left side of the plot, the trematodes *Aptorchis elegans* (4), *Aptorchis glandularis* (5), *Aptorchis kuchlingi* (6), *Buckarootrema minutum* (21), and *Pretestis laticaecum* (113) show a clear association with the families Chelidae and Carettochelyidae, which are predominantly distributed in Oceania, forming a compact cluster.

The results also show that trematodes associated with native species tend to cluster more centrally and to the left side of the projection, occurring near families such as Chelidae, Podocnemididae, and Geoemydidae.

### Platyhelminthes : Monogenea and cestoda

**Fig. 6:**
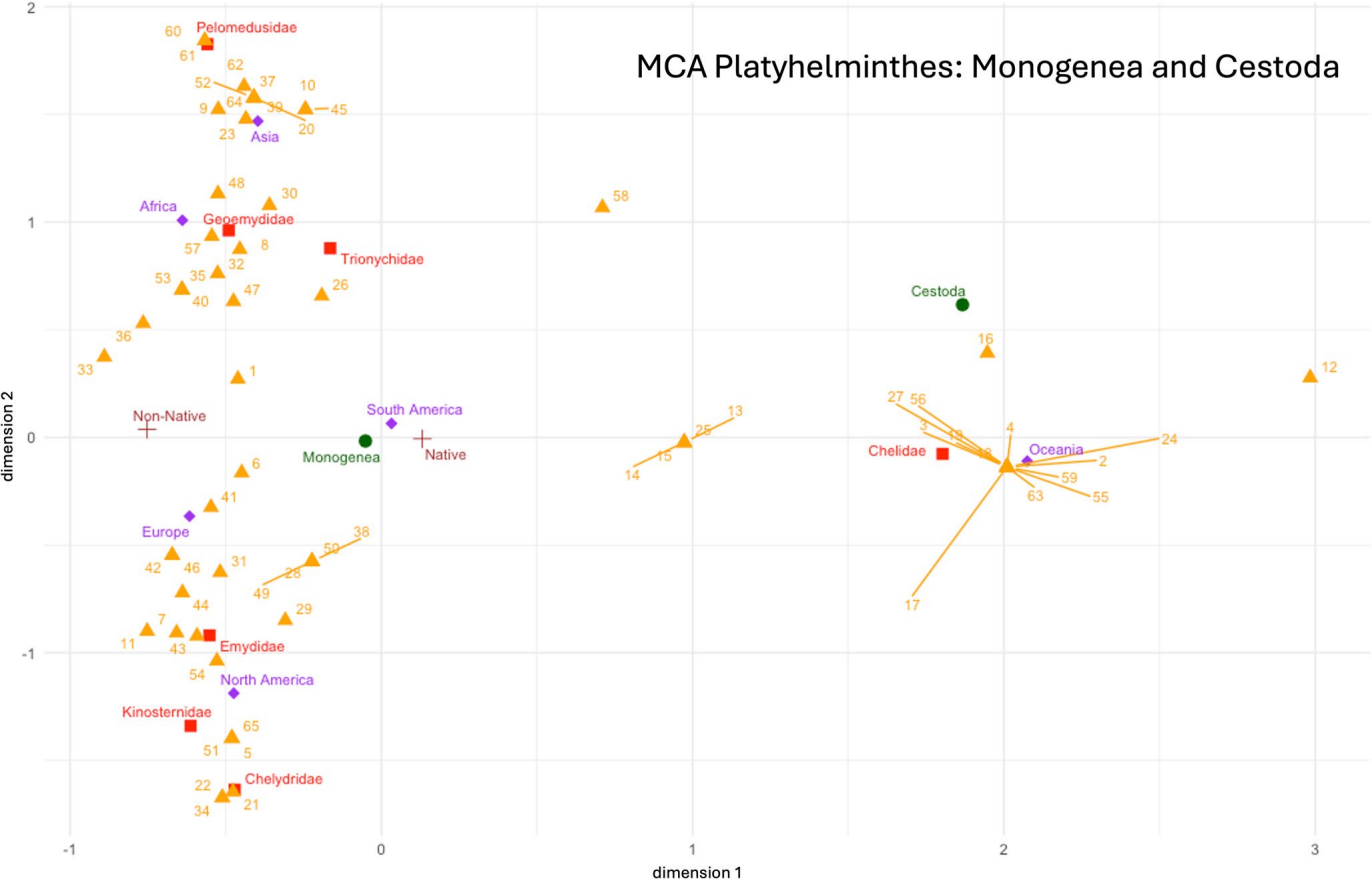
Two-dimensional representation of the results of the Multiple Correspondence Analysis (MCA) applied to the occurrence data of Platyhelminthes, Monogenea and Cestoda (numbered variables), in freshwater turtle hosts. Numbers are listed in Supplementary Materials 3. Host species are categorised according to taxonomic family (red), continent of origin (purple), and occurrence status (pink: Native or Non-Native). Legend: Orange numbers correspond to the different trematode morphotypes identified in the analysed turtles; red squares represent the taxonomic families of the turtle hosts; purple diamonds indicate the continents of origin of the host species; pink crosses refer to the occurrence status of the host species within the study area (Native or Non-Native).

Based on the results obtained through the MCA, a clear separation of Platyhelminthes is observed, particularly between representatives of the classes regions of origin, and occurrence status (Fig. 6).

The Monogenea show a marked proximity to *Uropolystomoides bourgati* (60), and *Uropolystomoides chabaudi* (61) within the family Pelomedusidae, situated near the vector corresponding to Asia, reflecting their association with Afro-Asian host species.

In the African region, Monogenea appear close to the families Geoemydidae and Trionychidae, indicating associations between this parasitic group and hosts belonging to these lineages. In Europe, the family Emydidae is the most represented, a pattern also observed in North America, where additional associations occur with the families Kinosternidae and Chelydridae. Within Chelydridae, notable parasites include *Polystomoidella oblonga* (21), *Polystomoidella whartoni* (22), and *Neopolystoma fentoni* (34).

In contrast, Cestoda are positioned farther from the centre of the MCA plot, showing a clear association with Oceania and the family Chelidae, including species such as *Ophiotaenia cohospes* (16).

Overall, Monogenea are predominantly associated with non-native species than Cestoda, which is mainly associated with Oceania, occurring mainly in native turtle species, reflecting distinct biogeographical and adaptive patterns between the two parasitic groups.

### Protozoa and Chromista

**Fig. 7:**
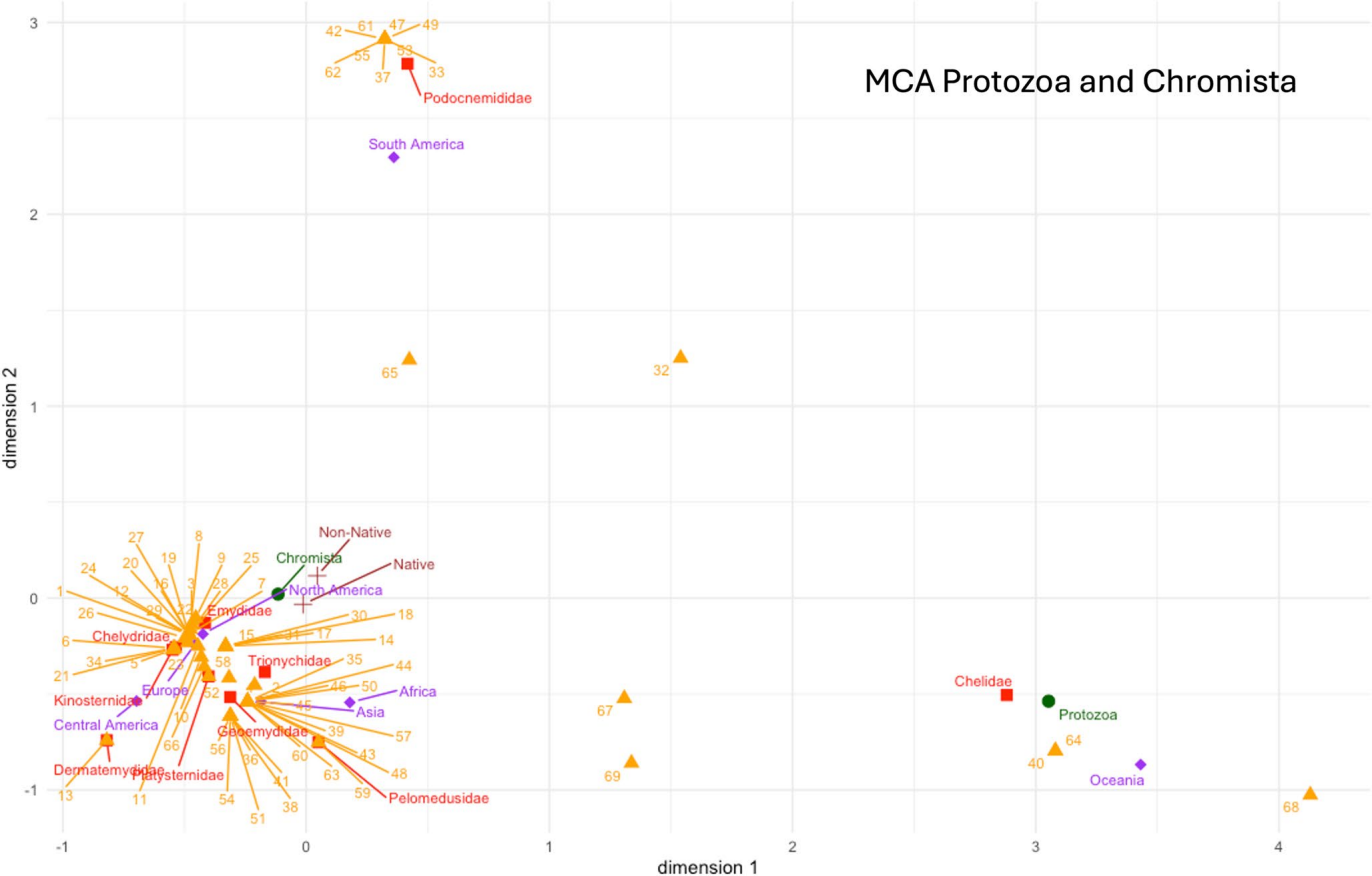
Two-dimensional representation of the results of the Multiple Correspondence Analysis (MCA) applied to the occurrence data of Protozoa and Chormista (numbered variables), in freshwater turtle hosts. Numbers are listed in Supplementary Materials 3. Host species are categorised according to taxonomic family (red), continent of origin (purple), and occurrence status (pink: Native or Non-Native). Legend: Orange numbers correspond to the different trematode morphotypes identified in the analysed turtles; red squares represent the taxonomic families of the turtle hosts; purple diamonds indicate the continents of origin of the host species; pink crosses refer to the occurrence status of the host species within the study area (Native or Non-Native).

The projection of variables in the MCA showed a clear separation between the parasitic groups Protozoa and Chromista (Fig. 7), indicating that these groups are associated with different distribution patterns. Chromista exhibit a broad distribution, occurring across all continents except Oceania, where no relevant associations are detected. In South America, the presence of this group is less pronounced; however, there is a clear relationship is observed with the family Podocnemididae with the protozoan species *Haemogregarina brasiliana* (37) and *Haemogregarina podocnemis* (53).

Across Europe, North America, and Central America, parasite association is evident among several turtle families, including Emydidae and Chelydridae, with *Eimeria chelydrae* (6) and *Eimeria mascoutini* (21); Kinosternidae and Platysternidae, with *Hemolivia pulcherrima* (66); and Dermatemydidae, with *Eimeria grayi* (13). These patterns suggest potential ecological and evolutionary similarities among hosts in these regions.

Conversely, turtle families from Africa and Asia display shared parasitic associations, as evidenced by *Aphanomyces sinensis* (2) and *Haemogregarina gangetica* (46). The family Pelomedusidae is notable for its exclusive association with the parasite *Haemogregarina sternotheri* (59).

In contrast, the Protozoa group is clearly associated with Oceania, particularly with the family Chelidae, represented by *Haemogregarina clelandi* (40) and *Haemoproteus chelodinae* (64). This reflects a distinct pattern of endemism and geographical specificity, differing markedly from that observed for Chromista.

### Nematoda and acanthocephala

**Fig. 8:**
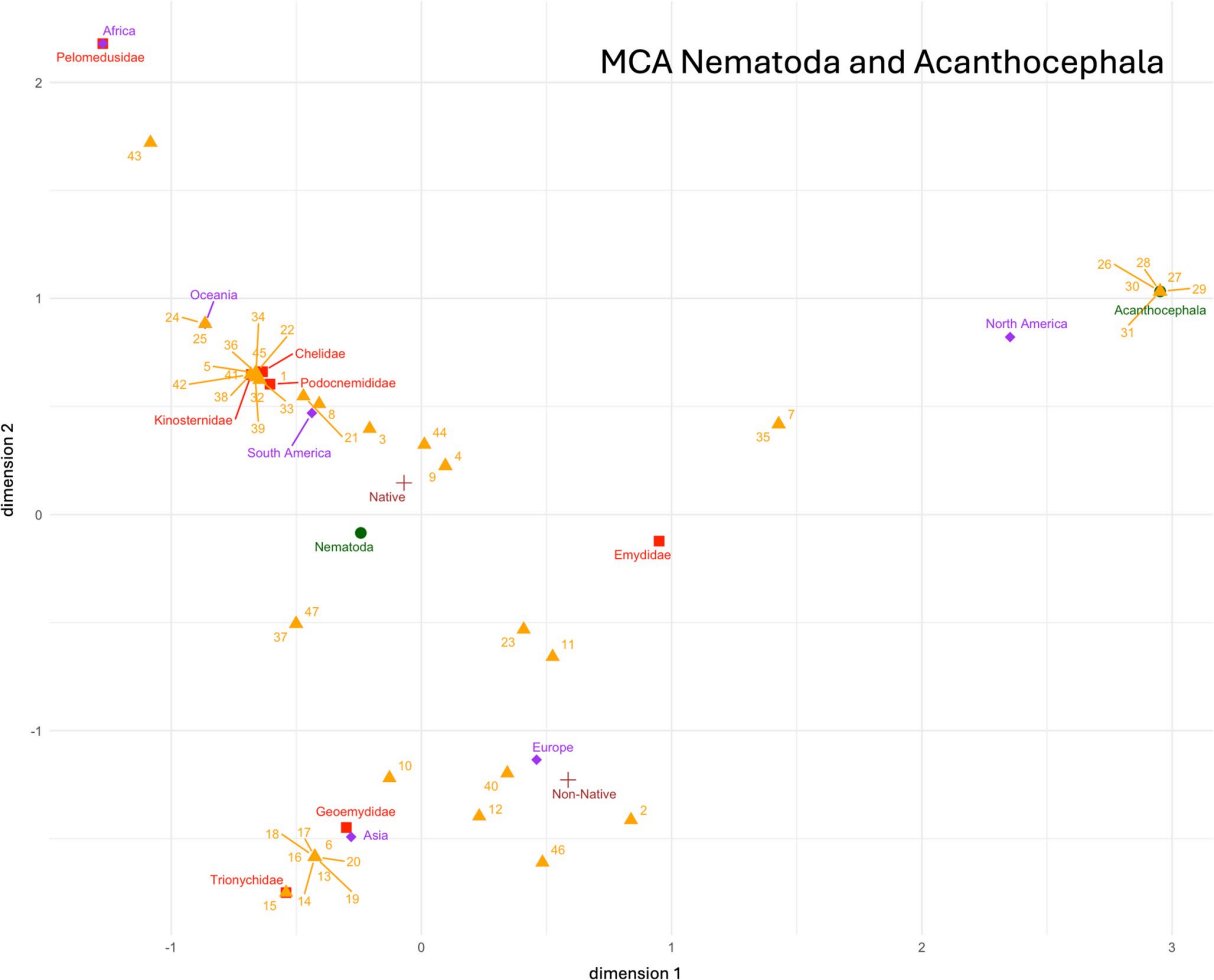
Two-dimensional representation of the results of the Multiple Correspondence Analysis (MCA) applied to the occurrence data of Nematoda and Acanthocephala (numbered variables), in freshwater turtle hosts. Numbers are listed in Supplementary Materials 3. Host species are categorised according to taxonomic family (red), continent of origin (purple), and occurrence status (pink: Native or Non-Native). Legend: Orange numbers correspond to the different trematode morphotypes identified in the analysed turtles; red squares represent the taxonomic families of the turtle hosts; purple diamonds indicate the continents of origin of the host species; pink crosses refer to the occurrence status of the host species within the study area (Native or Non-Native).

The MCA analysis reveals a clear separation between the groups Nematoda and Acanthocephala, indicating distinct patterns of association between parasites, turtle families, and geographic regions (Fig. 8).

The Acanthocephala group appears clearly isolated within the multidimensional space, being exclusively associated with the species *Neoechinorhynchus chrysemydis* (26), *N. emydis* (27), *N. emyditoides* (28), *N. pseudemydis* (29), *N. schmidti* (30), and *N. stunkardi* (31), all of which are reported from North America.

In contrast, Nematoda display a broader geographic and host taxonomic distribution, encompassing multiple turtle families across several continents. In Africa, the nematode *Hedruris pendula* (23) has been recorded in hosts of the family Pelomedusidae. In Oceania and South America, nematode sharing is observed among the families Chelidae, Podocnemididae, and Kinosternidae, predominantly involving native hosts. In Europe, the species *Serpinema microcephalus* (40) and *Angusticaecum holopterum* (2) are recorded. Finally, in Asia, the families Geoemydidae and Trionychidae stand out, with the latter being specifically associated with the *Falcaustra japonensis* (15).

Non-native hosts are more closely associated with Europe and Asia, belonging mainly to the families Geoemydidae and Trionychidae. Overall, the MCA plot demonstrates that nematodes exhibit broad ecological and geographical plasticity, whereas acanthocephalans display a restricted distribution limited to North America.

### Ectoparasites

**Fig. 9:**
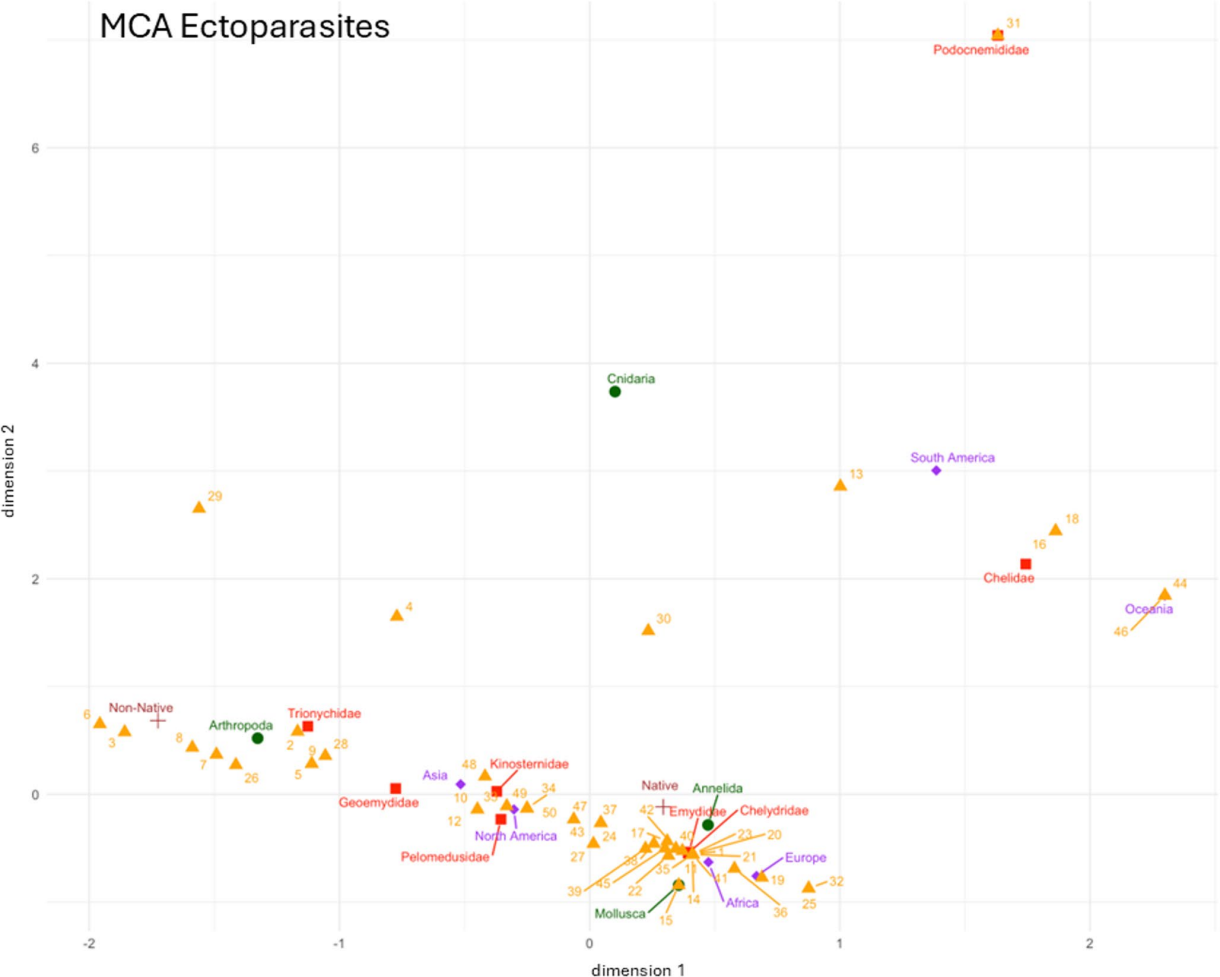
Two-dimensional representation of the results of the Multiple Correspondence Analysis (MCA) applied to the occurrence data of Ectoparasites (numbered variables), in freshwater turtle hosts. Numbers are listed in Supplementary Materials 3. Host species are categorised according to taxonomic family (red), continent of origin (purple), and occurrence status (pink: Native or Non-Native). Legend: Orange numbers correspond to the different trematode morphotypes identified in the analysed turtles; red squares represent the taxonomic families of the turtle hosts; purple diamonds indicate the continents of origin of the host species; pink crosses refer to the occurrence status of the host species within the study area (Native or Non-Native).

Based on the MCA applied to the records of ectoparasites (Fig. 9), the family Podocnemididae appears isolated, positioned in the upper right quadrant of the plot, and is strongly associated with *Myxidium turturibus* (31). This cluster is also related to South America and, to a lesser extent, to the family Chelidae, which is linked to the species *Haementeria brasiliensis* (16), *Helobdella adiastola* (18), *Placobdelloides bancrofti* (44), and *Placobdelloides octostriata* (46), all restricted to Oceania.

The phylum Annelida includes *Erpobdella punctata*(14), *Ferrissia californica* (15), *Helobdella modesta* (21), and *Helobdella octatestisaca* (22), while the phylum Mollusca includes *Placobdella ali* (35) and *Placobdella parasitca* (42). These parasites are closely associated with the families Emydidae and Chelydridae, with records reported mainly from Europe and Africa, mostly involving native freshwater turtle hosts.

Finally, the phylum Cnidaria is represented by a single isolated point in the upper central area of the plot, indicating low number of records with the other parasite groups recorded.

### General parasite groups pattern

Multivariate analyses of the major parasitic groups indicate that the composition of parasite communities in FT seems to be determined by the phylogenetic relation of the hosts and their biogeographic distribution. Trematodes form well-defined clusters, with clear associations among Asian species (Trionychidae, Geoemydidae), South American taxa (Podocnemididae), and North American/European hosts (Emydidae, Chelydridae, Kinosternidae). Within Platyhelminthes, there is a marked separation between Monogenea and Cestoda: Monogenea are primarily associated with non-native species and are distributed across Africa, Europe, and North America, whereas Cestoda are concentrated in Oceania and are linked exclusively to native families such as Chelidae and Carettochelyidae.

A similarly pronounced segregation is observed between Protozoa and Chromista. Chromista, which include vectors of haemogregarine parasites, occur widely across several continents and show strong associations with Podocnemididae, Emydidae, and Chelydridae. In contrast, Protozoa display a highly endemic pattern in Oceania, being associated exclusively with Chelidae. Among Nematoda and Acanthocephala, the distinction is equally clear: Acanthocephala are restricted to North America, while Nematoda show a broader and more flexible distribution, infecting host species in Africa, Oceania, South America, and Asia.

Ectoparasites also display well-defined regional clusters, with Podocnemididae forming an isolated group in South America and Chelidae associated with a distinct cluster in Oceania, while annelid and molluscan ectoparasites are more frequently associated with Emydidae and Chelydridae, particularly in European and African records.

## Discussion

Our results show clear patterns in how parasite communities in FTs are structured, shaped by phylogenetic relations, geography, and the introduction of non-native species. While regions such as North America, Asia, South America, and Europe show extensive parasite sharing, often driven by non-native species like *T. scripta*, Africa and Oceania have mostly endemic parasite communities restricted to native turtles.

The low proportion of publications addressing parasites and pathogens in reptiles is largely attributable to the fact that research on this group has been overwhelmingly dominated by studies on helminths, which mostly represent the endemic parasitofauna of healthy hosts rather than emerging epidemic infections (Johnson and Paull 2011). In reptiles, no specific helminth parasite has been identified as particularly dominant, showing that there is no single high-impact parasite capable of drawing major scientific attention or driving more focused research (Johnson and Paull 2011).

Most of the available records have been published after the beginning of the 21 st century, with a particularly evident increase following the SARS-CoV-2 pandemic, which profoundly reshaped both social and scientific perceptions of the role of wildlife in the emergence of zoonotic diseases. This global event led to a substantial reinforcement of interest and scientific output related to infectious diseases in wild fauna (Ternova et al. 2022).

The distribution pattern highlights a concentration of scientific effort in regions with higher diversity and abundance of FTs, consistent with previous reviews of wildlife diseases, where most studies also originated from North America, Asia, and Europe (Tompkins et al. 2015; Wiethoelter et al. 2015). In contrast, Africa remains comparatively understudied, likely reflecting a historical neglect of its chelonian diversity by both scientific and conservation communities, despite representing approximately 15% of global turtle species (Luiselli et al. 2021). As noted by Luiselli et al. (2021), although disease is recognized as a major global threat, it is currently considered of limited relevance for West African species, likely reflecting the low level of research attention in the region.

This global analysis of parasite sharing among FT demonstrates that biogeography, host phylogeny, and the introduction of non-native species are significant factors influencing observed patterns. Closely related genera such as *Trachemys*, *Graptemys*, and *Pseudemys* exhibited a high degree of parasite overlap, as they belong to the same family and share overlapping native habitats. In Europe, a dense network of interactions is evident between native and non-native turtles, with *T. scripta* emerging as a central parasitic connector. This overlap is largely driven by the breaking of historic biogeographic barriers, namely due to the increase in global trade, which has accelerated species translocations and the movement of their parasites across regions (Hulme, 2009). Parasite transmission between native and non-native freshwater turtle species in natural populations had already been documented in the Iberian Peninsula, particularly from *M. leprosa* and *E. orbicularis* to *T. scripta elegans* (Hidalgo-Vila et al. 2009; Oi et al. 2012; Verneau et al. 2025). *T. scripta elegans* became the most widely traded pet turtle during the second half of the twentieth century (Verneau et al. 2011). Together with other North American turtles from the genera *Apalone*, *Graptemys*, *Pseudemys* and *Chrysemys*, millions of individuals were exported worldwide, especially to Asian and European markets (Verneau et al. 2011). *T. scripta* is now listed among the 100 most harmful invasive species globally (Lowe et al., 2000) and competes directly with the native turtles, e.g. *E. orbicularis* and *M. leprosa* in Europe, for food, basking sites, and nesting areas, posing a significant threat to their conservation (Cadi and Joly, 2003; Polo-Cavia et al. 2009). Despite this, the consequences of parasite sharing between invasive and native turtles remain poorly understood, underscoring the need for continued parasitological surveillance of both native and non-native species to assess transmission risks and ecological impacts.

In contrast, in Oceania, parasite connections occur exclusively among native species, indicating a cohesive endemic community. The absence of parasite sharing between native and non-native turtles is consistent with the ecological vulnerability of insular systems and may reflect strong parasite divergence, limiting transmission between native and non-native lineages. Insular ecosystems are known to be particularly sensitive to invasions, with impacts often amplified compared to continental systems (Vitousek et al., 1988; Bellard et al. 2016). This vulnerability is further reinforced by the ecological and evolutionary characteristics of islands, including high endemism, small population sizes, reduced biodiversity, and taxonomic disharmony (Losos and Ricklefs 2009; Simberloff, 2000; Williamson, 1981; Cushman 1995), as well as the limited adaptive capacity of native species (Tershy et al. 2015). Collectively, these factors help explain the restricted integration of introduced turtles and the absence of parasite exchange with native hosts in Oceania (Vitousek et al., 1988; Tershy et al. 2015). In Oceania, parasite sharing occurs exclusively among native species, reflecting strong biogeographic isolation of host lineages and a limited capacity for introduced turtles to integrate into local communities or participate in parasite transmission.

In Asia, parasite sharing is extensive among multiple native genera but also includes significant parasite sharing between non-native and native species, indicating a high degree of parasitological permeability. These findings align with Neely et al. (2017), who examined 40 turtles across four species of Geoemydidae and recovered fifteen parasite species, including nematodes, trematodes, and one leech, across multiple host genera. This highlights substantial native parasite sharing among native species. Moreover, the documentation of thirteen new parasite–host associations demonstrate a strong capacity of parasites to colonise novel hosts, likely facilitated by the extreme confinement conditions of the wildlife trade, where thousands of turtles are housed together for extended periods (Neely et al. 2017). For example, the leech *Placobdelloides stellapapillosa* was recorded in *S. crassicollis* for the first time, illustrating ectoparasite transfer across species of different origins (Neely et al. 2017). In Africa, parasite sharing is almost entirely restricted to native species, with no evidence of interactions involving introduced turtles. However, Africa is also the continent with the fewest analyzed articles and consequently, these findings could be partly explained by the limited availability of data.

Across the Americas, parasitological networks are highly interconnected, with North America structured mainly by genera such as *Trachemys*, *Graptemys*, and *Pseudemys*, and South America centred on *Phrynops* and *Acanthochelys*. Interactions between non-native and native hosts in South America remain limited, likely reflecting the lower number of turtle introductions on the continent (GBIF, 2025). These patterns mirror the exceptional FT diversity of the Nearctic and Neotropical regions, recognised among the world’s major centres of turtle diversity alongside the Oriental Region (Bour 2007). North America represents an important region for FT diversity, particularly for families such as Emydidae, Kinosternidae, and Chelydridae (Rhodin et al. 2018), whereas South American turtle diversity is dominated by Pleurodira, particularly the families Chelidae and Podocnemididae (Rhodin et al. 2018), it also includes Neotropical cryptodiran taxa such as the genus Rhinoclemmys, which is widely distributed across northern South America (IUCN, 2023). The broad distribution of genera such as *Trachemys* and *Kinosternon* across both regions likely enhances ecological connectivity and parasite overlap. Overall, parasite diversity in the Americas appears strongly shaped by native turtle lineages and host biogeography (Bour 2007), while introduced species may facilitate parasite exchange between native and non-native hosts, acting as epidemiological bridges (Verneau et al. 2011).

Our results shows that parasite communities in FTs are mainly shaped by the evolutionary history of their hosts and by their geographic distribution. These patterns result from the interaction between phylogenetic conservatism and biogeographic constraints, which determine the realized host specificity (Wells and Clark 2019). Phylogenetic conservatism is widely recognised as a major force structuring parasite biodiversity (Agosta et al. 2010; Poulin 2014), leading closely related host species to share similar parasite faunas with comparable richness (Poulin 2014). The clustering of Trematoda by host families from Asia, South America, and North America/Europe reflects this strong phylogenetic specificity (Poulin et al. 2011). Colonization of new hosts is limited by physiological and ecological barriers, requiring compatibility between parasite and host (Wells and Clark 2019).

Similarly, the concentration of Cestoda in Oceania, linked exclusively to native families (Chelidae and Carettochelyidae), suggests a strong coevolutionary link or, more likely, ecological fitting via resource tracking (Agosta et al. 2010), where parasites use pre-existing traits to exploit hosts that share similar ecological characteristics. Ecological fitting allows parasites to colonize new hosts without needing new evolutionary changes (Agosta et al. 2010).

From a biogeographic perspective, parasite richness closely follows local host richness, meaning that spatial patterns of parasite diversity tend to mirror those of their hosts (Poulin 2014). This is visible in ectoparasites, which showed well-defined regional clusters (Podocnemididae in South America and Chelidae in Oceania), suggesting that host distribution contributes to the structuring of host–parasite assemblages at regional scales (Wells and Clark 2019). The restriction of Acanthocephala to North America is explained by geographic barriers that limit the parasite’s realized niche (Wells and Clark 2019). Although some acanthocephalans in fish can infect hosts from very different families (showing potential generalism) (Poulin et al. 2011), their restricted distribution here suggests that environmental conditions and local host availability determine realized specificity in each region (Wells and Clark 2019).

The wide and flexible distribution of Nematoda indicates a higher degree of generalism and lower phylogenetic specificity compared with more restricted groups (Poulin et al. 2011). Host specificity is not fixed, and environmental conditions can strongly influence realized specificity (Wells and Clark 2019). Generalist parasites can exploit hosts from different families (Poulin et al. 2011). The strong contrast between the distributions of Nematoda and those of Acanthocephala and Trematoda shows that parasite groups differ in specialization and phenotypic plasticity (Agosta et al. 2010).

The preferential association of Monogenea with non-native hosts across Africa, Europe, and North America highlights the importance of contemporary host shifting (Wells and Clark 2019). Introduced species can act as carriers of parasites or alter local interactions, affecting the realized host specificity (Wells and Clark 2019). Therefore, biological invasions increase opportunities for ecological fitting, enabling parasites to colonize new hosts using pre-existing traits (Agosta et al. 2010). This process is central to emerging infectious diseases, as parasites can infect hosts with no prior evolutionary association.

In summary, the regional and phylogenetic patterns observed do not reflect strict co-divergence but rather a macroevolutionary mosaic (Agosta et al. 2010). The structure of parasite communities in FTs results from historical inheritance (phylogenetic conservatism in Trematoda and Cestoda) combined with ecological and geographic factors (host availability and non-native species introductions) that promote ecological fitting across spatial scales (Agosta et al. 2010). Because parasite richness is tightly linked to host richness, any factor that alters host diversity, such as biogeography, will inevitably influence parasite diversity (Poulin 2014).

It is important to highlight the bias in the available data. For example, in Africa, the seemingly low parasite diversity may reflect the limited number of studies conducted on FTs. In contrast, species such as *T. scripta* show higher parasite records due to their wide distribution and the scientific attention they receive (GBIF, 2025). In North America, the reported high parasite diversity is largely associated with the large number of studies carried out in the region. Similarly, *P. sinensis* has received greater attention in Asia due to its relevance as a food source. These factors underscore the need to consider sampling bias when interpreting patterns of parasite diversity and sharing.

## Conclusions

This global assessment demonstrates that the structure of parasite communities in freshwater turtles is strongly influenced by host phylogeny, biogeographic history, and the presence of non-native species. Regions such as North America, Asia, and South America show the most diverse and interconnected parasitological networks, whereas insular systems like Oceania maintain highly cohesive and endemic parasite communities, with no evidence of parasite exchange involving introduced turtles. These patterns underscore the role of evolutionary isolation and regional species composition in shaping distinct host–parasite assemblages.

In Europe, the non-native *T. scripta* acts as a key epidemiological bridge, connecting native and non-native parasite networks through its widespread dispersal via the pet trade. Although the ecological consequences of parasite spillover and spillback remain poorly understood, the overlap between native and introduced parasites highlights the importance of ongoing monitoring. Most FT parasites are native helminths with low zoonotic potential, but the increasing recognition of wildlife as a source of emerging infectious diseases emphasizes the need to integrate chelonian parasitology into broader health and conservation strategies. Captive trade conditions, where thousands of FT are kept together, increase the risk of pathogen amplification and cross-species transmission, potentially affecting both wildlife and public health. Together, these results underline the need for further studies on the parasitic fauna of FT, in order to achieve stricter control of non-native species introductions and to better understand and mitigate the ecological and epidemiological consequences of parasite sharing in FT.

## Supplementary Information

Below is the link to the electronic supplementary material.

Supplementary Material 1 (DOCX 13.5 KB)

Supplementary Material 2 (XLSX 22.5 KB)

Supplementary Material 3 (XLSX 214 KB)

## Data Availability

This is a original data.
