# Supplementary material for: Global patterns of parasite sharing among freshwater turtles
Source: Parasitol Res. 2026 Jun 2;125(1):70. doi: 10.1007/s00436-026-08702-5 (PMC13260133; doi:10.1007/s00436-026-08702-5)
Supplement: Supplementary file 1 — Supplementary Material 1 (DOCX 13.5 KB) [file 436_2026_8702_MOESM1_ESM.docx]

# Supplementary Materials 1

Keywords used in the literature search in  Google Scholar, Web of Science and Scopus:

“freshwater turtle” AND “disease” AND “bacteria”; “freshwater turtle” AND “disease” AND “virus”; “freshwater turtle” AND “disease” AND “fungus”; “freshwater turtle” AND “disease” AND “fungi”; “freshwater turtle” AND “disease” AND “parasite”; “freshwater turtle” AND “disease” AND “protozoa”; “pond turtle” AND “disease” AND “bacteria”; “pond turtle” AND “disease” AND “virus”; “pond turtle” AND “disease” AND “fungus”; “pond turtle” AND “disease” AND “fungi”; “pond turtle” AND “disease” AND “parasite”; “pond turtle” AND “disease” AND “protozoa”; “terrapin” AND “disease” AND “bacteria”; “terrapin” AND “disease” AND “virus”; “terrapin” AND “disease” AND “fungus”; “terrapin” AND “disease” AND “fungi”; “terrapin” AND “disease” AND “parasite”; “terrapin” AND “disease” AND “protozoa”; “freshwater turtle” AND “parasite”; “freshwater turtle” AND “protozoa”; “pond turtle” AND “parasite”; “pond turtle” AND “protozoa”; “terrapin” AND “parasite”; “terrapin” AND “protozoa”.
